# Solvent-Assisted Laser Desorption Flexible Microtube Plasma Mass Spectrometry for Direct Analysis of Dried Samples on Paper

**DOI:** 10.1021/acs.analchem.3c03009

**Published:** 2023-10-30

**Authors:** Marcos Bouza, Norman Ahlmann, Juan F. García-Reyes, Joachim Franzke

**Affiliations:** † Analytical Chemistry Research Group, Department of Physical and Analytical Chemistry, University of Jaén, Campus Las Lagunillas, 23071 Jaén, Spain; ‡ ISAS—Leibniz Institut für Analytische Wissenschaften, Bunsen-Kirchhoff-Str. 11, 44139 Dortmund, Germany

## Abstract

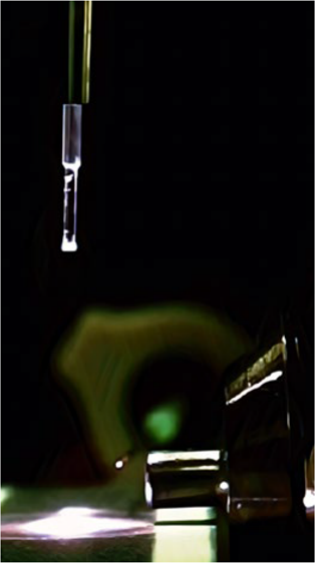

The present study investigated the potential for solvent-assisted laser desorption coupled with flexible microtube plasma ionization mass spectrometry (SALD-FμTP-MS) as a rapid analytical technique for direct analysis of surface-deposited samples. Paper was used as the demonstrative substrate, and an infrared hand-held laser was employed for sample desorption, aiming to explore cost-effective sampling and analysis methods. SALD-FμTP-MS offers several advantages, particularly for biofluid analysis, including affordability, the ability to analyze low sample volumes (<10 μL), expanded chemical coverage, sample and substrate stability, and in situ analysis and high throughput potential. The optimization process involved exploring the use of viscous solvents with high boiling points as liquid matrices. This approach aimed to enhance desorption and ionization efficiencies. Ethylene glycol (EG) was identified as a suitable solvent, which not only improved sensitivity but also ensured substrate stability during analysis. Furthermore, the addition of cosolvents such as acetonitrile/water (1:1) and ethyl acetate further enhanced sensitivity and reproducibility for a standard solution containing amphetamine, imazalil, and cholesterol. Optimized conditions for reproducible and sensitive analysis were determined as 1000 ms of laser exposure time using a 1 μL solvent mixture of 60% EG and 40% acetonitrile (ACN)/water (1:1). A mixture of 60% EG and 40% ACN/water (1:1) resulted in signal enhancements and relative standard deviations of 12, 20, and 13% for the evaluated standards, respectively. The applicability of SALD-FμTP-MS was further evaluated by successfully analyzing food, water, and biological samples, highlighting the potential of SALD-FμTP-MS analysis, particularly for thermolabile and polarity diverse compounds.

## Introduction

Paper, as a substrate, has emerged as a valuable sampling support due to its advantageous features, including easy transportation and rapid and cost-effective sampling. Among the platforms utilizing paper, microfluidic paper-based analytical devices (μPADs) stand out as a notable example.^1^ The versatile design and cost-effectiveness of μPADs have boosted their utilization in areas like food safety,^2^ environmental analyses,^3^ and diagnostics.^4^ It is also a powerful tool to help implementing diagnostic approaches in countries with inadequate infrastructures.^5^ However, the detection on μPADs commonly involves the utilization of electrochemical methods,^6^ colorimetric assays,^7^ or naked-eye observation.^8^ Unfortunately, these detection methods present inherent challenges that limit the applicability of μPADs, such as limited sensitivity, a lack of multiplexed analysis capability, and time-consuming analysis procedures.

To address these limitations, the integration of mass spectrometry (MS) has proven highly promising, offering advantages such as high sensitivity, low volume requirements, multiplexed analysis capabilities, automation potential, and accurate identifications. The inclusion of portable mass spectrometers has unlocked the use of MS in point-of-care (POC) analysis for resource and resource-limited regions.^9^ More importantly, μPADs and paper-based POC platforms can readily leverage the benefits provided by MS. One notable example is paper spray (PS),^10^ which has been successfully utilized for the analysis of biofluids and immunoassays,^11,12^ as well as small and large molecules.^13,14^ Despite the straightforward nature of applying a voltage to a triangular-cut paper, the broader applicability of PS has been restricted due to inherent sensitivity limitations for direct analysis of compounds with complex matrices and challenges associated with coupling PS to PADs, which entail additional sample treatment and time-consuming reactions.^15,16^ Ambient MS, such as desorption electrospray ionization (DESI)^17^ and liquid microjunction surface sampling probe (LMJ-SSP),^18^ has also drawn attention for directly analyzing samples deposited on paper. However, these ion sources could suffer from potential differences in analyte extraction, ionization suppression problems, and limitations regarding analyte polarity due to their electrospray ionization (ESI)-like nature.

The samples could also be directly desorbed from a surface. Laser desorption is the preferred option and, depending on the application, allows for mass spectrometry imaging (MSI).^19^ The use of laser desorption has been proposed as a perfect match for atmospheric pressure sampling, using ultraviolet (UV), and infrared (IR) lasers.^20^ However, these lasers require expensive and intricate setups, hampering on-site analysis and complicating the analysis of samples deposited on a substrate like paper. Laser diode thermal desorption^21^ has also been proposed to desorb samples from surfaces, with the use of a hand-held diode laser as an alternative for eased on-site analysis while maintaining cheap approaches.^22,23^

Postionization for laser desorption and MSI helps to ionize the ejected plume of material more efficiently.^20^ Different postionization means, such as laser postionization for matrix-assisted laser desorption ionization (MALDI-2),^24^ ESI postionization (MALDESI, LAESI),^25,26^ and photionization,^27^ have been proposed. The simplicity and adaptability of dielectric barrier discharges (DBD) helped to a rapid development as an alternative postionization ion source.^28,29^ Ion sources like DBD ionization,^30^ low-temperature plasma,^31^ active capillary plasma ionization (ACaPI),^32^ and its commercial counterpart, soft ionization by a chemical reaction in transfer (SICRIT),^33^ as well as the flexible microtube plasma (FμTP),^34^ have been used for applications ranging from imaging to environmental monitoring and forensics.^35−37^

Here, we propose a novel method based on solvent-assisted laser desorption (SALD) in combination with FμTP coupled with MS for the analysis of samples deposited on paper in less than 5 s. A hand-held IR laser (940 nm) is used for the sample desorption. The different parts of the coupling were selected to ensure easy access, keeping in mind the key goals of affordability and ease of implementation and operation; a comprehensive cost analysis of the setup is presented in the Supporting Information. The method was optimized to favor analyte desorption while preserving the paper and sample integrity using a solvent. The nature of the solvent and the laser-FμTP parameters were evaluated and optimized using analytical standards (amphetamine, imazalil, and cholesterol). To demonstrate the capabilities of SALD-FμTP-MS, different types of samples (food, tap water, and biofluids) deposited on paper were analyzed. To the best of our knowledge, this is the first attempt to use a solvent as an extractive/desorption matrix for the analysis of laser-desorbed samples from thermolabile substrates.

## Experimental Section

### Chemicals and Materials

Acetonitrile (ACN), butylene glycol (BG), ethylene glycol (EG), isopropyl alcohol (IPA), methanol (MeOH), propylene glycol (PG), and water (LC-MS grade) were purchased from Sigma-Aldrich (Madrid, Spain). Whatman No. 1 paper was obtained from Cytiva (Buckinghamshire, UK) through Sigma-Aldrich (Madrid, Spain). The MALDI matrices, including 2,5-dihydroxybenzoic acid (2,5-DHB), α-cyano-4-hydroxycinnamic acid (CHCA), and 3-nitrobenzonitrile (3-NBN), were also acquired from Sigma-Aldrich (Madrid, Spain). Analytical standards of amphetamine, cholesterol, cocaine, codeine, imazalil, and methamphetamine as well as human plasma were purchased from Sigma-Aldrich (Madrid, Spain). All solutions were prepared by mixing MeOH and water (1:1) to obtain the desired concentrations. For optimizing SALD-FμTP-MS, a solution containing 50 μM amphetamine, imazalil, and cholesterol was used.

### Sample Preparation

The substrate used for samples was Whatman No. 1 paper patterned by using a Xerox Phaser 8560J wax printer, mimicking the pattern shown in Figure S1. After wax printing, the paper was subjected to heating at 120 °C for 60 s followed by cooling to room temperature.^38^ Thus, it permitted the diffusion of the wax through the paper, creating confined sampling spots. The diameter of the sampling spot before heating was 4 mm. The patterned paper was used to control the sampling spot and avoid diffusion of the liquid sample and the liquid matrices. Prior to sample deposition, the opposite side of the sampling area was drawn with graphite from a 6B pencil obtained from a local store. The addition of the graphite layer improved the paper’s capacity to absorb laser energy by having a colored surface, as white surfaces exhibit limited effectiveness in absorbing IR irradiation.

Five microliters of each standard and sample, including the spiked oral fluid and the plasma, was deposited on the prepared Whatman No. 1 paper substrate. In the case of blood, 20 μL was deposited on the paper. Additional information regarding the biological samples and the specific sampling protocol can be found in the Supporting Information. All deposited samples were allowed to dry for 1 h before proceeding with SALD-FμTP-MS analysis.

### SALD- FμTP-MS Platform

**Figure 1:**
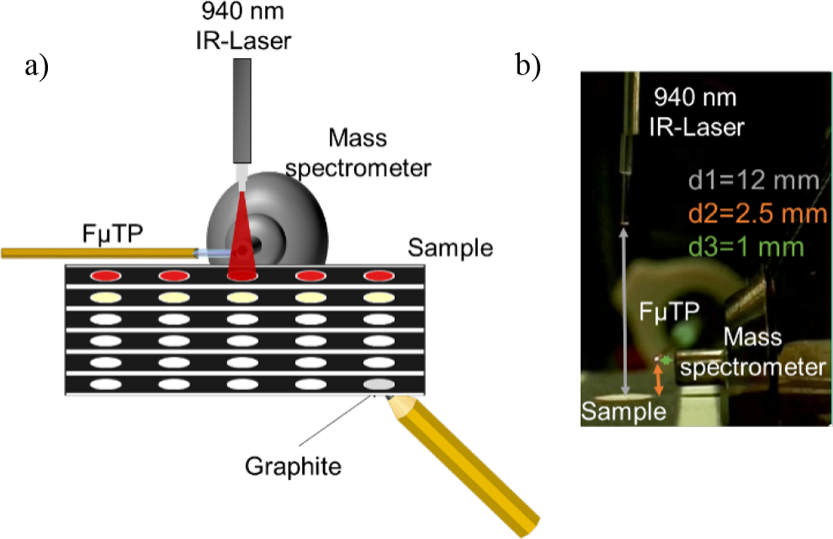
(a) Schematic representation of the SALD-FμTP-MS final setup. (b) Photograph displaying the setup with the optimized distances utilized for the analysis. The distance used were d1 = 12 mm, d2 = 2.5 mm, and d3 = 1 mm.

Figure 1a illustrates the schematic of the home-built SALD-FμTP source. The setup consists of a hand-held diode IR laser (iLase, Biolase Tech, Irvine, CA, USA) operating at 940 nm for sample desorption, a FμTP (previously described)^34^ serving as a postionization source, and a holder for positioning the wax-patterned paper. The final configurations and distances between the components are depicted in Figure 1b.

Initially designed for dental surgical applications, the IR laser incorporates a rechargeable battery with a 1 h lifetime. A disposable 400 μm fiber optic tip was employed to direct the laser beam to the ablation zone. The laser operates in three modes: continuous wave (CW), pseudocontinuous wave modes CP1 (0.1 ms on/0.2 ms off), and CP2 (1.0 ms on/1.0 ms off). The laser maximum power is 3 W, and for these experiments, it was operated in CW mode at 3 W. Typically, the laser is manually operated, but to overcome the challenges associated with hand operation (i.e., irreproducibility spot to spot), a digital timer controller (EZM-3735, EMKO elektronics, Bursa, Turkey) was utilized. This controller not only allowed precise control of the exposure time (0.01 s precision) but also provided the contact-closure voltage necessary for autonomous laser operation, enhancing the reproducibility and reliability of the results.

The FμTP was constructed by using a flexible polyimide-coated fused silica capillary with an inner diameter of 250 μm and an outer diameter of 360 μm. A tungsten wire with a diameter of 100 μm was positioned 5 mm from the end of the capillary as the unique electrode. A 100 mL/min helium (N5.0) flow rate was maintained to sustain the plasma. The ignition and sustainment of the plasma were achieved by applying a square wave high voltage using an in-house-built square-wave generator. The generator operated at a fixed frequency of 20 kHz with a slope of 60 V/ns and was connected to a tungsten wire. The end of the FμTP capillary was positioned at 90° with respect to the mass spectrometer inlet, separate 6 mm, to facilitate the interaction of the plasma with the desorbed analytes.

The experiments were conducted by using a Thermo Finnigan LTQ linear ion trap mass spectrometer (Thermo Scientific, San José, CA, USA). The following parameters were employed for positive ion mode: a capillary inlet temperature of 300 °C, a capillary voltage of 18 V, a tube lens voltage of 100 V, 1 microscan, and a maximum injection time of 200 ms. For negative ion mode, the parameters were as follows: a capillary inlet temperature of 300 °C, a capillary voltage of −10 V, a tube lens voltage of −16 V, 1 microscan, and a maximum injection time of 200 ms. Tandem MS analysis using collision-induced dissociation (CID) was utilized for analyte identification, employing a 2 Da isolation window and 25 normalized energies for CID experiments.

#### Safety Considerations

The employed continuous-wave diode laser is of laser safety class 4. Safety precautions must be taken when working with free beams of such lasers by wearing protective goggles.

## Results and Discussion

### Optimization of the Analysis Parameters for SALD-FμTP-MS

**Figure 2:**
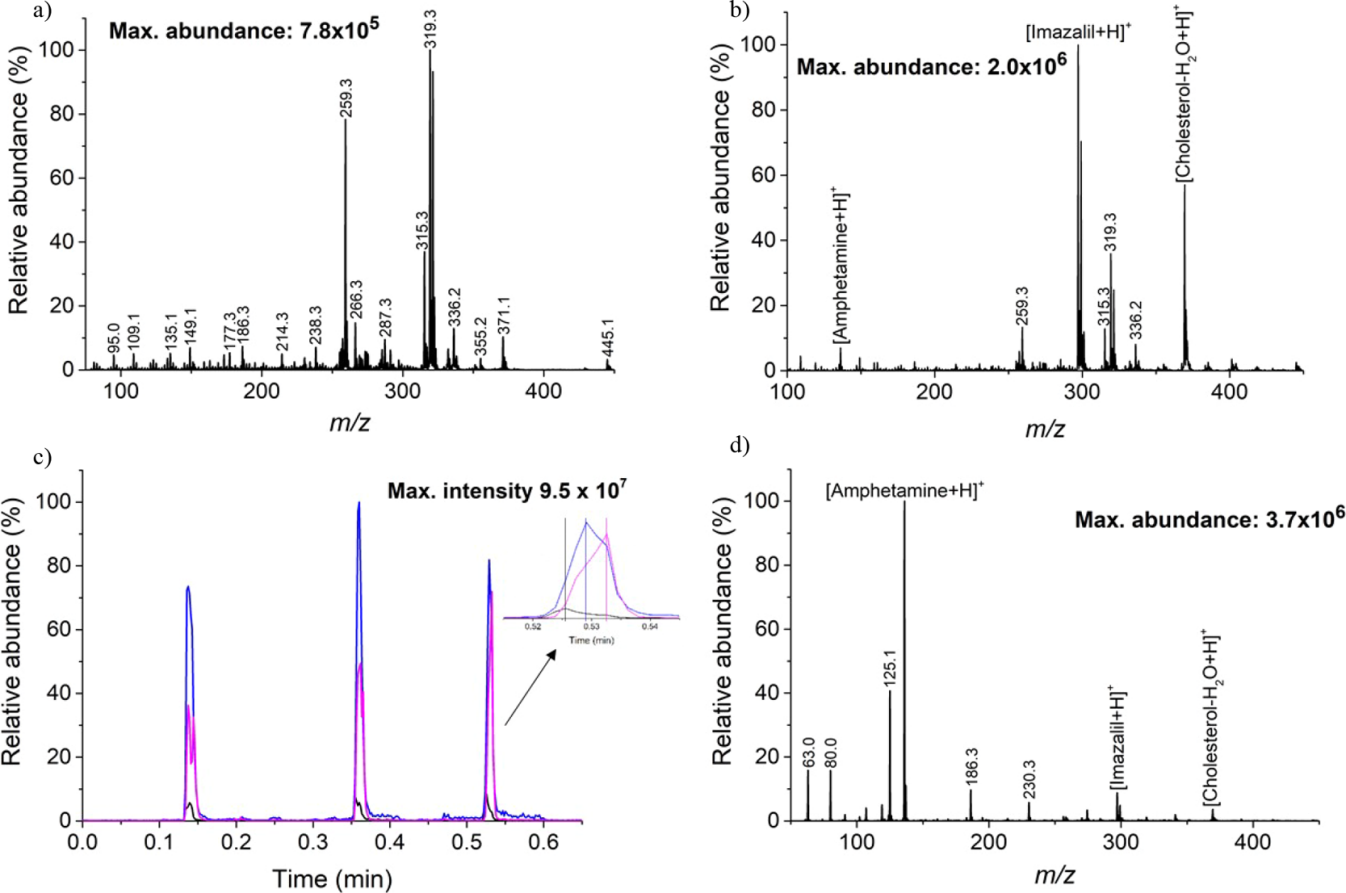
Comprehensive analysis and evaluation of the results obtained under different conditions using LD-FμTP-MS for the analysis of 250 picomoles of amphetamine, imazalil, and cholesterol deposited on patterned paper. (a) Mass spectrum obtained from LD-FμTP-MS analysis of a sample (250 picomoles) deposited on white paper without graphite. (b) Mass spectrum obtained from LD-FμTP-MS analysis of the same sample deposited on white paper with graphite applied on the backside. (c) Extractive ion chromatogram (EIC) of three replicates in three different spots for imazalil (blue trace), cholesterol (pink trace), and amphetamine (black trace); in the figure, the desorption order of the compounds. (d) Mass spectrum obtained from SALD-FμTP-MS analysis of the sample deposited on white paper with graphite on the backside and EG as the desorbing solvent.

Initially, we tested the analysis of samples deposited on paper using LD-FμTP-MS for direct desorption using a hand-held IR laser, avoiding the liquid matrix. However, as anticipated, the white paper did not absorb the laser energy, resulting in the detection of background ions generated by the FμTP (Figure 2a). Previous studies have utilized graphite as a substitute for the matrix in MALDI or as a laser energy-absorbing substrate on surfaces like paper or thin-layer chromatography plates.^39−41^ In our case, when graphite was deposited on top of the sample, the paper quickly burned when using continuous mode (CW) at power levels ranging from 1 to 3 W. However, when a thin paper such as Whatman No. 1 paper with a thickness of 180 μm was used, the graphite could be drawn on the backside of the paper, preventing contact with the sample and enhancing the laser exposure time (>1 s) for improved desorption (Figure 2b). The resulting mass spectra permitted the detection of 250 picomoles of amphetamine, imazalil, and cholesterol (*m*/*z* = 136.2, 297.2, and 369.4, respectively). However, imazalil and cholesterol had abundances 2 orders of magnitude higher when compared to amphetamine. The analysis of three different spots demonstrated relative standard deviations (RSD) of 12% for amphetamine and imazalil and 14% for cholesterol (Figure 2c). One interesting observation was the gradual desorption of the compounds from the paper potentially linked to their boiling points, with amphetamine (203 °C) desorbing first followed by imazalil (347 °C) and cholesterol (360 °C) (inset, Figure 2c). In the case of IR laser, the desorption is strictly related to the temperature gradient promoted by the laser.

Postionization helped to drastically improve the ionization efficiency of the laser drastically. When the IR laser was used as desorption and ionization means barely ionized the compounds, and when ionization occurred, the signal was 3 or 4 orders of magnitude lower than FμTP (Figure S2). The laser is known to have poor ionization efficiency,^42^ so the observed ions could be the product of assisted ionization by a heated mass spectrometer inlet.^43,44^

To ensure the sample and substrate stability while avoiding excessive heating, we investigated alternative approaches. Liquid support matrices, as proposed for MALDI and AP-MALDI before,^45−47^ were evaluated. The liquid matrices could enhance intershoot reproducibility and facilitate the extraction of polar molecules from the paper. Viscous liquids with high boiling points were preferred to promote reproducible extraction and heat dispersion, thereby preventing substrate burning. EG, a polar solvent with a boiling point of 197 °C and a higher density of 1.12 g/mL, was identified as a suitable solvent used previously for liquid AP-MALDI.^46^ EG demonstrated improved capabilities for the analysis of samples deposited on paper, allowing for better detection of polar molecules while preserving the integrity of the paper during laser operation (Figure 2d). The signal for amphetamine was enhanced by 25 times, while the abundances of imazalil and cholesterol were reduced by approximately 5 times. The solvent aided in desorbing the sample and facilitated the transmission of polar molecules by forming a spray plume after laser irradiation, as shown in the slow motion video in the Supporting Information. The highest abundances were observed for SALD-FμTP when the postionization ion source was placed between the sample and the mass spectrometer inlet (Figure 1b). These observations support the mechanism proposed by Koch et al.^48^ since the sample in liquid AP-MALDI first is ablated but requires an external source, in their case, heating to favor later ionization. Moreover, SALD-FμTP-MS does not require a high voltage applied to the plate or the high-temperature transfer tube to enhance ionization as in the case of MALDI.

As shown in Figure 1b, the distances between the fiber optic of the laser and the sample (d1), the paper and the mass spectrometer inlet (d2), and the FμTP and the mass spectrometer inlet (d3) were optimized to maximize different aspects. The fiber optic was placed at a distance of 12 mm from the paper to desorb the entire area of the spotted sample as we observed that shorter distances promoted rapid burning of the paper and resulted in lower signals (data not shown). The patterned paper used wax; to avoid polymeric contamination, the paper should not touch the heated inlet. Lastly, the plasma should be placed between the desorbed material and the mass spectrometer inlet, facilitating the interaction with the spray and potential desorbed neutrals.

**Figure 3:**
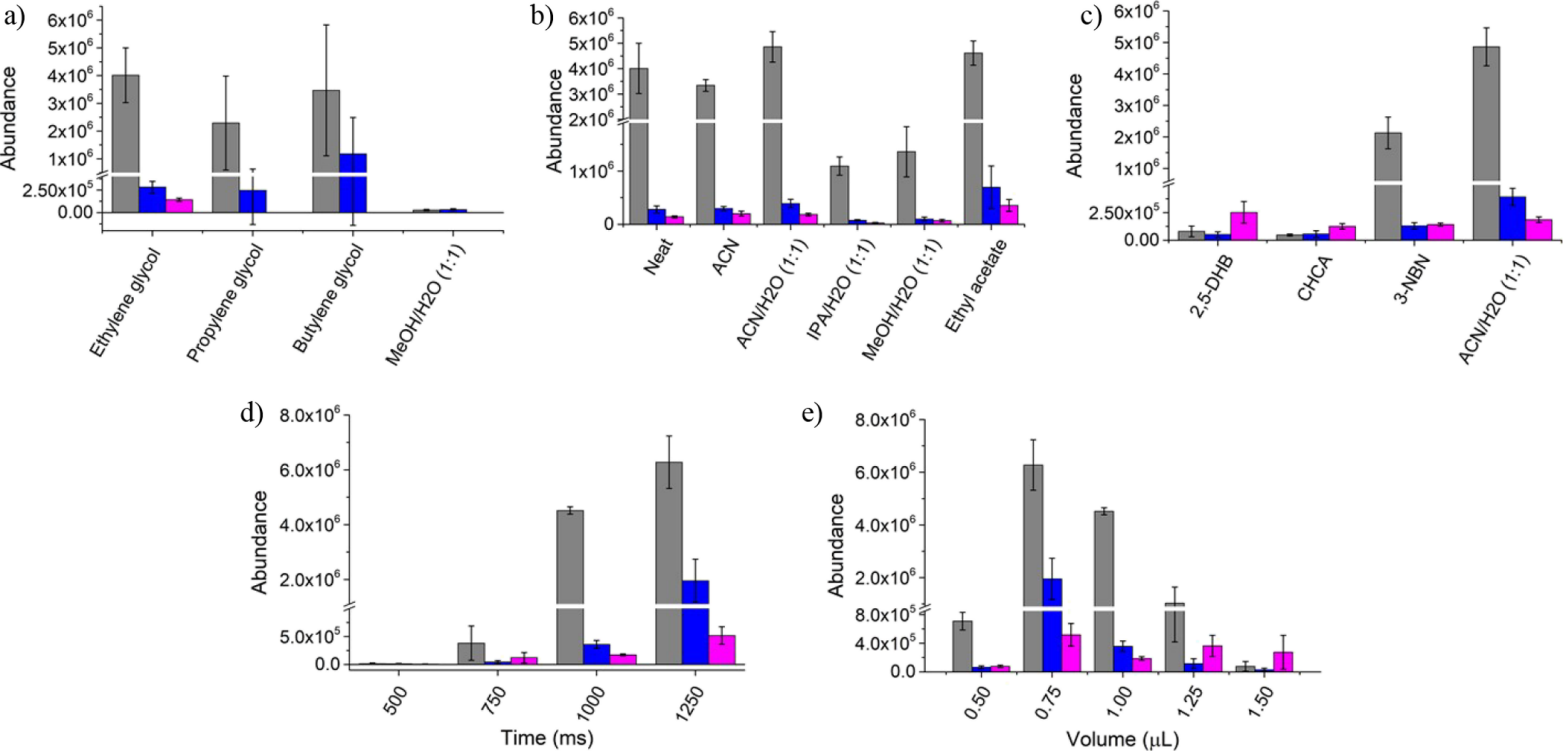
Optimization of SALD-FμTP-MS parameters for the analysis of 250 picomoles of amphetamine, imazalil, and cholesterol deposited on patterned paper by investigating the (a) solvent effect, (b) cosolvent effect, (c) MALDI-like matrix influence, (d) laser exposition time, and (e) solvent volume. The bars corresponded to amphetamine (gray), imazalil (blue), and cholesterol (pink).

The evaluation and optimization of different parameters in SALD were conducted to improve the desorption and ionization efficiencies. Viscous solvents with high boiling points, such as EG, PG, and BG, were tested. While they helped to desorb and ionize the evaluated compounds, PG and BG showed poorer reproducibility with high RSD for the detected compounds (Figure 3a). The mass spectra of PG and BG (Figures S3 and b) showed lower abundances and dominant signals for *m*/*z* 135.0 ([2PG + H]^+^) and *m*/*z* 147.1 ([2BG + H]^+^). Additionally, a mixture of MeOH/water (1:1) was also evaluated, but it resulted in low abundances and very rapid, uneven solvent evaporation, leading to poor reproducibility with an RSD exceeding 30% and the nondetection of cholesterol (Figure S3c). Neat EG showed a 22% of RSD between analyses, so the addition of a cosolvent was explored to enhance sensitivity and reproducibility.

Cosolvents such as ACN/water (1:1) and ethyl acetate were added to EG in a 40% concentration, following similar trends as in liquid AP-MALDI.^49^ ACN/water (1:1) provided moderate signal enhancement for the compounds with RSDs of 12, 20, and 13% for amphetamine, imazalil, and cholesterol, respectively. Ethyl acetate showed higher signal enhancement but was associated with higher levels of irreproducibility (RSD: 10, 57, and 31%). Other cosolvents like MeOH/water and IPA/water did not show good performance for the analysis (Figure 3b). Methanol has been shown as poorly efficient for the desorption due to the low boiling point and the problems with the temperature distribution. In the case of IPA, the boiling point is similar to that of ACN but is a compound with lower polarity. Based on these results, a mixture of 60% EG and 40% ACN/water (1:1) was chosen for SALD-FμTP.

In contrast to liquid AP-MALDI and AP-MALDI, where the matrix plays a crucial role in ionization, the presence of matrices such as 2,5-DHB, CHCA, and 3-NBN was not beneficial for SALD-FμTP-MS. The matrices mixed with the liquid matrix suppressed or decreased the ionization of the target compounds (Figure 3c). As observed in Figure S4a and b, the mass spectra were dominated by *m*/*z* 125.1, [2EG + H]^+^, and no signal was observed at *m*/*z* 156 and 190 for the [M + H]^+^ ions of 2,5-DHB and CHCA, respectively. Although 3-NBN, a matrix used in matrix-assisted ionization^50^ and sonic-spray ionization,^51^ showed higher abundances compared to other matrices, it was still inferior to the EG and ACN/water (1:1) cosolvent.

The laser exposure time and volume of the liquid matrix were found to be correlated. Optimal laser exposure was achieved using 1 μL of the solvent volume. Shorter exposure times (500 and 750 ms) resulted in poor desorption and low abundances. Increasing the exposure time to 1000 ms allowed for complete solvent evaporation and reproducible analysis. Longer exposure times (1250 ms) led to higher abundances for high boiling point compounds at the expense of reproducibility (Figure 3d). The solvent volume followed a similar trend when 1000 ms of exposure time was used, with insufficient extraction and low abundances for volumes that were too low (e.g., 500 μL), and inefficient desorption and ionization for volumes that were too high (e.g., 1.25 and 1.50 μL). In the case where the volume was lower than the required volume for the selected exposure time (0.75 μL), the signals for the three standards increased but the RSD also increased. The sample was better desorbed, and the temperature of the paper increased, but when the solvent evaporated, the analysis became more erratic, affecting reproducibility. The optimal conditions for reproducible and sensitive analysis were 1000 ms of exposure time using 1 μL of solvent (Figure 3e).

The presence of a liquid matrix was beneficial for the analysis of thermolabile compounds. Ketones with increasing aliphatic chain lengths were evaluated (Table S1). The molecules dehydration was observed when no liquid matrix was used during the desorption (Figure S5a), except for 2-pentadecanone, which has a higher boiling point (293 °C), and its low polarity makes it a perfect match for plasma-based ionization. However, when the optimized liquid matrix was used, the dehydration of the ions was avoided, and the [M + H]^+^ ions were detected for all the evaluated ketones (Figure S5b).

Another option to limit the substrate overheating and destruction was to work in pulsed modes predefined in the hand-held IR laser.^23^ In the case of using liquid matrices, we did not observe desorption working in CP2 mode at 3 W and little efficiency in the case of the CP1 mode at 1.6 W (Figure S6).

SALD-FμTP-MS also worked in negative ion mode, as demonstrated by the detection of glycolic, lactic, and hippuric acid when 5 nmoles was deposited on the paper (Figure S7). However, the negative ion mode mass spectra showed multiple background signals that required further exploration to optimize the analysis in that mode.

#### Applications

##### Food Analysis

In the field of food analysis, there is a constant demand for new tools and methods that can simplify and accelerate the analysis of foodstuffs. Rapid analysis techniques with minimal or no sample preparation are crucial in taking prompt action to prevent potential public health issues. Traditional reference techniques such as gas chromatography and liquid chromatography (LC) often require lengthy and intricate sample pretreatment procedures to simplify the complex matrices present in food samples, which can lead to matrix effects and ion suppression.

As an alternative, ambient MS has emerged as a promising approach.^52^ Its key advantages include rapid analysis and the requirement for low sample volumes. As one such technique, we proposed SALD-FμTP-MS. It offers a versatile platform that can be easily adapted for high throughput analysis, eliminating the necessity for electrical contact to initiate the spray, a requirement prevalent in PS-MS. By creating a multispot pattern on a filter paper sheet (Figure S1) and utilizing a motor for movement in the *XY* directions, SALD-FμTP-MS becomes an ideal tool for rapid screening of liquid foodstuffs.

**Figure 4:**
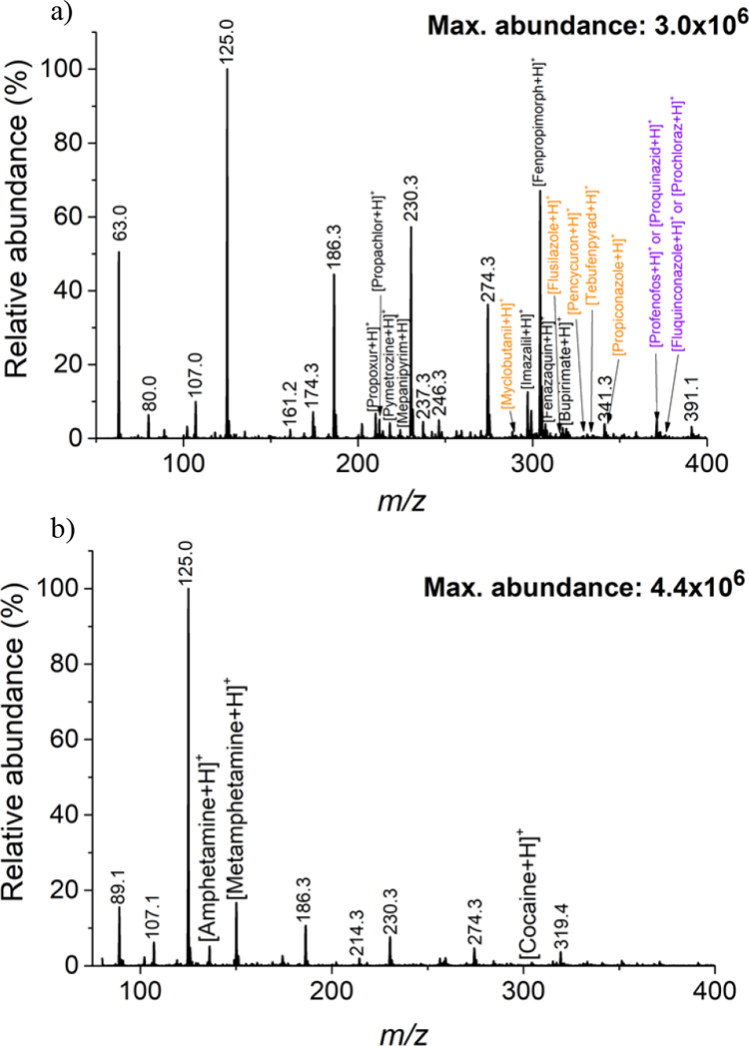
Mass spectra of an energy drink analyzed in (a) positive ion mode and (b) negative ion mode.

To illustrate the application of SALD-FμTP-MS, we conducted an analysis of a popular energy drink, Red Bull. Analyzing such beverages typically involves multiple pretreatment steps to address their complex matrices. In this case, we deposited 5 μL of the energy drink onto the paper and applied the optimized analysis procedure. Positive ion mode analysis (Figure 4a) revealed the presence of caffeine, niacinamide, and vitamin B6. Surprisingly, we did not detect any amino acids (such as taurine, phenylalanine, and lysine) that were previously detected using electrospray-based techniques.^53,54^ These small polar molecules are challenging to detach from the paper or ionize through FμTP. Figure S8 demonstrates that when we deposited 5 μL of a mixture of 8 amino acids at a concentration of 5 mM (25 nmoles) on the paper and analyzed it using SALD-FμTP-MS, some of the compounds were barely detected.

Switching to negative ion mode analysis (Figure 4b) provided complementary information, detecting lactic acid, niacin, 3-(2-hydroxyphenyl) propanoate, and lauric acid. However, due to the resolving power limitations of the ion trap used for analysis, it was not possible to differentiate other compounds such as resorcinol monoacetate or 2-methoxybenzoic acid, inositol, glucose, or fructose, and citric acid or isocitrate.

##### Water Analysis

Water analysis is of great importance due to the concern over the presence of contaminants in drinking water and its potentially harmful effects on both environmental and human health. Similar to food analysis, LC-MS is the most versatile tool for analyzing and characterizing residues of pharmaceuticals, personal care products, pesticides, and other pollutants that may be present in water.^55^ However, LC-MS analysis often involves lengthy extraction processes and consumes large amounts of solvents. Here, we highlight the significant features of ambient MS, including speed, low sample and solvent consumption, and another crucial property: portability. The ability to perform on-site analysis reduces the need to transport and store large amounts of samples.

**Figure 5:**
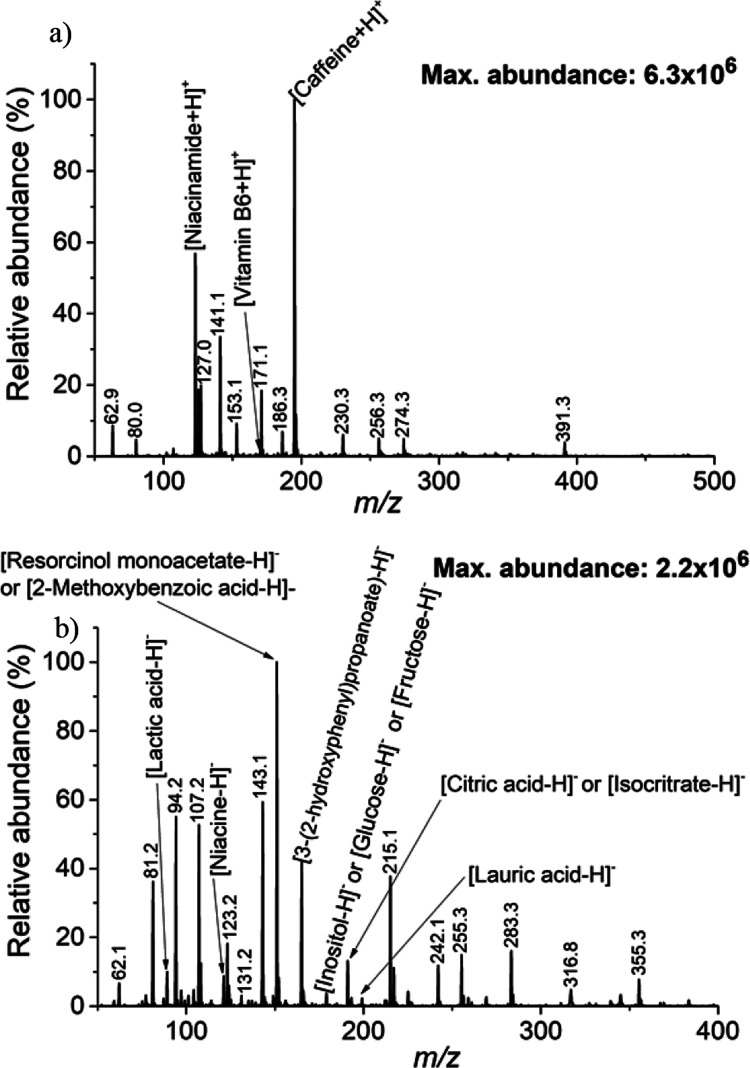
Mass spectra of an energy drink analyzed in (a) positive ion mode and (b) negative ion mode.

The use of paper as a substrate offers a rapid platform for sampling minimal amounts of sample while also facilitating transportation and storage. In this study, we evaluated two different scenarios. First, a mixture of 31 pesticides (listed in Table S2) was spiked in tap water at a concentration of 500 pg/μL each. Subsequently, 5 μL of the sample was deposited on a patterned paper, resulting in a final mass of 2.5 ng for each pesticide. The ion species detected are labeled in Figure 5a. Out of the 31 compounds, SALD-FμTP-MS enabled the detection of 11 compounds at high abundances (green in Table S2), 5 compounds at low abundances (yellow in Table S2), and 2 groups of isobaric compounds (purple in Table S2). However, 14 compounds were not detected at the evaluated concentration.

In the second scenario, amphetamine, methamphetamine, and cocaine were spiked in tap water at a final concentration of 100 pg/μL each. Following the same pattern, 5 μL of the sample was deposited on patterned Whatman No. 1 paper, resulting in 500 pg of each drug on each spot. As depicted in Figure 5b, all three drugs (amphetamine, methamphetamine, and cocaine) were readily detected at *m*/*z* 136.1, 150.1, and 304.2, respectively, representing the [M + H]^+^ ions. Tandem MS analysis revealed diagnostic fragments for each species (highlighted in red in Figure S9), confirming the presence of the compounds and the capability to detect lower concentrations in samples with clean matrices, such as water.

##### Biofluid Analysis

Dried biofluid spots have been extensively utilized in clinical laboratories, particularly in newborn screening, for many years. Paper serves as an excellent substrate for sampling small volumes of biofluids, such as blood, plasma, and urine, allowing for secure storage over long periods. However, conventional analysis methods often require punching a specific area of the paper, leading to potential irreproducibility, and time-consuming extraction steps become necessary. PS has previously been proposed as an ambient MS alternative for directly analyzing biofluid samples deposited on paper.^13,11^ However, the complex matrices of biofluids can result in ion suppression, which sometimes hampers PS–MS analysis.^56^ It is important to highlight that PS–MS, functioning as a spray-based ion source, is typically constrained to the analysis of polar molecules under standard operational conditions. In contrast, SALD-FμTP-MS offers several advantages, featuring an expanded chemical coverage that facilitates the analysis of both polar and low-polar molecules, such as amphetamine and cholesterol. It also holds promise for reducing matrix effects compared to ESI-like sources. atmospheric pressure chemical ionization (APCI) techniques have been acknowledged for their reduced susceptibility to ion suppression.^57,58^ However, it is important to recognize that SALD-FμTP-MS may potentially encounter ion suppression effects, although still predicted to be less pronounced than those in PS-MS. Nevertheless, the investigation of these effects falls beyond the scope of the current study; yet, it constitutes an integral component of our planned future research in this field.

In this study, we spiked different drugs into oral fluid and plasma and characterized the blood to evaluate the performance of SALD-FμTP-MS.

**Figure 6:**
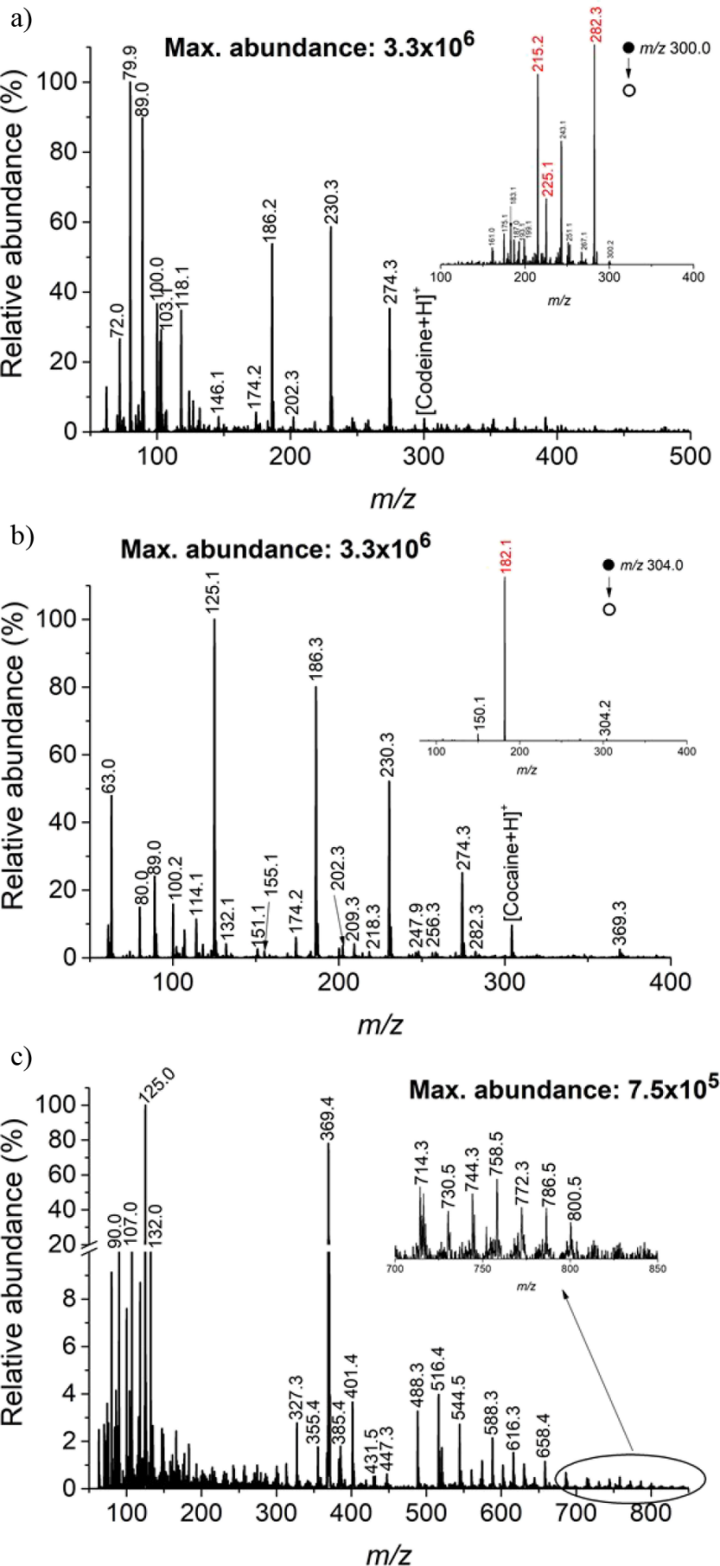
Mass spectra of (a) oral fluid spiked with 2 ng/μL of codeine, (b) plasma spiked with 2 ng/μL of cocaine, and (c) blood. The insets show the tandem MS spectra for (a) codeine and (b) cocaine and (c) the zoom-in of *m*/*z* range 700–850.

First, the oral fluid was examined. As depicted in Figure 6a, SALD-FμTP-MS easily detected codeine at a concentration of 2 ng/μL, corresponding to 10 ng deposited on the paper. The identification of codeine was confirmed by diagnostic fragment ion species at *m*/*z* 282.3, 225.1, and 215.2, obtained through tandem MS analysis performed for *m*/*z* 300 (highlighted in red on Figure 6a inset). Even at lower concentration levels, as low as 100 pg/μL (500 pg deposited on the paper) of codeine in oral fluid, detection and identification were achieved via tandem MS analysis (Figure S10a). Although oral fluid is a complex matrix that can lead to matrix effects,^59^ promising results were obtained without specific method optimization.

Next, human plasma was evaluated. The sample was spiked with 2 ng/μL cocaine, leading to 10 ng on the paper. Figure 6b shows the [M + H]^+^ ion for cocaine at *m*/*z* 304.2, along with other signals from the plasma and desorption solvent. The inset in Figure 6b presents the tandem MS spectrum of cocaine with a prominent intensity for the diagnostic ion species fragment at *m*/*z* 182. Even at a concentration of 500 pg/μL of cocaine in plasma, a clean tandem MS spectrum was obtained (Figure S10b).

Lastly, bovine blood was analyzed. Since blood is a colored sample, graphite was not required to promote desorption during SALD-FμTP-MS analysis. Twenty microliters of the sample was deposited using 10 μL of EG-ACN-water as the desorption and analysis solvent. It was observed that, although the signal proportions were higher, the signals, in general, were reduced due to the complex matrix of blood (Figure 6c). The main species tentatively identified from the analysis were lipids. In previous studies, FμTP has been proposed as a suitable method for detecting neutral lipids like cholesterol.^60,36^ However, the lack of a high-resolution mass spectrometer limited the analysis. In addition to *m*/*z* 125.0, the dominant signal corresponded to *m*/*z* 369.4, previously attributed to cholesterol. Potential oxidation products at *m*/*z* 385.4 and 401.4 were also detected. The oxidation may have occurred during plasma ionization, or it could be an inherent product of the blood, as *m*/*z* 401.4 could be attributed to 7-dehydrocholesterol, previously detected with APCI in a different biological sample.^61^ The detection of species with a higher molecular weight (*m*/*z* 450–800), potentially attributed to glycerophospholipids (GP), paves the way for further research in this area. The observed spray during the desorption step could be responsible for the ionization of polar lipids in the mass spectrometer inlet. The inlet capillary was maintained at 300 °C and, as observed by Michael et al.,^42^ higher temperatures in this region should enhance the detection of GPs. Table S3 provides the tentative annotations for the low-resolution MS analysis of bovine blood using the LIPID MAPS database with a delta of ±0.1 *m*/*z*. Further experiments employing a high-resolution mass spectrometer should be conducted to provide reliable annotations.

## Conclusions

SALD-FμTP-MS is a promising technique for the rapid and cost-effective analysis of various samples deposited on paper. The successful detection of sample composition, pesticides, and drugs in different samples and matrices underscores the potential of the technique. It offers significant advantages over conventional methods by allowing direct analysis of paper with low sample and liquid matrix volumes while permitting a wider chemical coverage (polar and low-polar molecules analysis).

The methodology employed in SALD-FμTP-MS utilizes the high boiling point of the liquid matrix to desorb the sample and ensure a homogeneous heat distribution within the sample spot. By incorporating cosolvents like acetonitrile or ethyl acetate into EG, improved desorption and the formation of the necessary spray for postionization are achieved. However, further refinement and development are required to optimize the methodology for specific applications.

The results showed that the potential portability and in situ analysis capabilities of SALD-FμTP-MS should be further investigated to enable rapid on-site monitoring and POC analysis. The experience gained from working with an ion trap mass spectrometer should facilitate the transition to analyzing samples with portable linear ion trap mass spectrometers.

Future research efforts should be focused on enhancing the sensitivity and selectivity of SALD-FμTP-MS by optimizing the ionization process, exploring different paper substrates and colored matrices, and employing high-resolution MS for more accurate compound identification. Moreover, expanding the application of SALD-FμTP-MS to areas such as pharmaceutical analysis and forensic sciences holds great potential and would be of significant interest.

## Supporting Information Available

Wax pattern model for printing; laser ionization in SALD-FμTP-MS analysis; influence of different liquid matrices; influence of MALDI matrices; impact of the liquid matrix on the analyte stability; efficiency of the laser pulse mode; SALD-FμTP-MS negative ion mode analysis; SALD-FμTP-MS analysis of amino acids; list of pesticides spiked and detected; tandem MS analysis of spiked compounds; tandem MS of lower concentrations of codeine and cocaine spiked in biofluids; and tentative identifications of lipids in bovine blood (PDF)
Solvent aided in desorbing the sample and facilitated the transmission of polar molecules by forming a spray plume after laser irradiation (MP4)
