# Supplementary material for: Solvent-Assisted Laser Desorption Flexible Microtube Plasma Mass Spectrometry for Direct Analysis of Dried Samples on Paper
Source: Anal Chem. 2023 Oct 30;95(50):18370–8. doi: 10.1021/acs.analchem.3c03009 (PMC10733904; doi:10.1021/acs.analchem.3c03009)
Supplement: Supplementary file 1 — ac3c03009_si_001.pdf [file ac3c03009_si_001.pdf]

Supporting information

Solvent-Assisted Laser Desorption Flexible Microtube Plasma Mass Spectrometry for Direct Analysis of Dried Samples on Paper

Marcos Bouza*[1], Norman Ahlmann[2], Juan F. García-Reyes[1] and Joachim Franzke[2]

[1]Analytical Chemistry Research Group, Department of Physical and Analytical Chemistry, University of Jaén, Campus Las Lagunillas, 23071 Jaén, Spain.

[2]ISAS—Leibniz Institut für Analytische Wissenschaften, Bunsen-Kirchhoff-Str. 11, 44139 Dortmund, Germany.

*Corresponding authors: Marcos Bouza, email: mbouza@ujaen.es, phone: +34 953 21 2758,

Table of Contents

Cost of SALD-FμTP-MS operation......S4

Biologial samples......S5

Figure S1. Wax pattern paper model for printing. The wax pattern was printed on Whatman No. 1 paper, with each spot initially having a diameter of 4 mm before undergoing thermal treatment. The total width of the pattern stripe is 6 mm. The use of the wax printer allowed for design flexibility, facilitating the creation of multispotted arrays. This design feature paves the way for potential automation of the sampling and analysis processes, ultimately enabling high throughput analysis......S6

Figure S1. Mass spectrum depicting the ionization potential achieved through laser desorption. The influence of discharge gas presence was evaluated by comparing the absence (0 mL/min He) and presence (100 mL/min He) of gas flow......S7

Figure S2. Mass spectra acquired through SALD-FμTP-MS analysis of the standard mixture sample deposited on white paper with graphite on the backside, utilizing different liquid matrices: a) propylene glycol (PG), b) butylene glycol (BG), and c) MeOH/water (1:1)......S8

Figure S3. Mass spectra obtained from SALD-FμTP-MS analysis of the sample deposited on white paper with graphite on the backside using various liquid matrices: a) 25 mg/mL of 2,5-dihydroxybenzoic acid (2,5-DHB) dissolved in 60% ethylene glycol (EG) and 40% acetonitrile (ACN)/water (1:1), b) 25 mg/mL of α-cyano-4-hydroxycinnamic acid (CHCA) dissolved in 60% EG and 40% ACN/water (1:1), c) 25 mg/mL of 3-nitrobenzonitrile (3-NBN) dissolved in 60% EG and 40% ACN/water (1:1), and d) 60% EG and 40% ACN/water (1:1) without any MALDI matrix added......S9

Figure S4. Impact of liquid matrix presence/absence on the fragmentation of the analytes using a mixture of seven ketones (2-hexanone, 2-heptanone, 2-octanone, 2-nonanone, 2-decanone, and 2-pentadecanone) at a concentration of 50 μM each in a MeOH/water (1:1) solution. a) SALD-FμTP-MS analysis without the liquid matrix, and b) SALD-FμTP-MS analysis with 60% ethylene glycol (EG) and 40% acetonitrile (ACN)/water (1:1) as the liquid matrix......S10

Table S1. Molecular weigth, *m/z* of the ions $[M+H]^+$ and $[M+H-H_2O]^+$, vapor pressure and boiling points of the seven evaluated ketones......S10

Figure S5. Mass spectrum obtained from SALD-FμTP-MS analysis in CP1 pseudo-continuous mode at 1.6 W using to desorb 250 picomoles of amphetamine, imazalil, and cholesterol deposited on patterned paper with graphite on the back of the paper. The liquid matrix utilized was a mixture of 60% EG and 40% ACN/water (1:1)......S11

Figure S6. Mass spectrum obtained from SALD-FμTP-MS analysis in negative ion mode of 5 nanomoles of glycolic acid, lactic acid, and hippuric acid. The desorption process was performed using a liquid matrix composed of 60% EG and 40% ACN/water (1:1) (black

trace), and without the liquid matrix (red trace)..S12

Figure S7. SALD-FµTP-MS analysis at the optimized conditions of 25 nanomoles of a mixture of 8 amino acids: alanine (Ala), serine (Ser), proline (Pro), threonine (Thr), glutamine (Gln), phenylalanine (Phe), arginine (Arg), and tyrosine (Tyr). The detected amino acids are highlighted in green, while the non-detected amino acids are highlighted in red in the side table..S13

Table S2. List of 31 pesticides spiked in the tap water sample and their corresponding mass spectrum in Figure 5a. The table includes highlighting for the detected compounds, compounds detected with low abundances, and potential isobars for the same *m/z*. The compounds in rows without highlighting indicate non-detected compounds......................S14

Figure S8. SALD-FµTP-MS Tandem MS mass spectra of the drugs spiked in the tap water sample collected in Figure 5b for a) amphetamine, b) methamphetamine, and c) cocaine..S16

Figure S9. SALD-FuTP-MS Tandem MS mass spectra analysis of a) 100 pg/µL codeine spiked in oral fluids, and b) 500 pg/µL cocaine spiked in human plasma..S17

Table S3. Candidates for tentative annotation of the *m/z* detected in bovine blood analysis as lipids. The candidates were determined using the LipidMaps annotation tool, considering the detected *m/z* values and selecting potential ion adducts $[M+H]^+$ and $[M+H-H_2O]^+$, since the ionization was carried out qith a plasma-based ion source, within a delta window of ±0.1 Da..S18

Cost of SALD-FμTP-MS operation

In terms of cost-effectiveness, it is noteworthy that SALD-FμTP-MS exhibits the potential to, at least, match PS-MS analysis in terms of economic feasibility. The laser utilized in this study, priced at approximately 3000€, not only exhibits user-friendliness but also demands minimal training for proficient operation. Furthermore, it should be underscored that cost reductions are reachable by employing diode lasers instead.

While the plasma operated in this work entails the use of a square-wave generator valued at approximately 10,000€, exceeding the expense associated with the DC power generator required for PS operation, it is important to highlight that the use of AC sinusoidal power supplies can yield comparable performance levels at significantly reduced costs, typically falling below 500€. Additionally, the construction of the FμTP ion source itself can be achieved for less than 50€, and helium consumption remains minimal (100 mL/min), further amenable to cost optimization by reducing the operation flow.

Another important cost-saving attribute of SALD-FμTP-MS is the low solvent requirements, necessitating merely 1 μL, in contrast to the 10 to 20 μL typically mandated for PS-MS analyses. This substantial reduction in solvent consumption contributes to a diminished operational cost per sample.

Biological samples

Oral fluid samples were collected from a healthy non-smoker male volunteer in our laboratory. After rinsing his mouth with water, 2 to 3 mL of oral fluid was collected by having the volunteer spit into a 15 mL Falcon tube. The collected sample was spiked with the analyte and vortexed for 20 seconds. Subsequently, the samples were stored at -20ºC until analysis.

The blood samples used in our experiments are bovine blood, procured from a pet shop and typically employed as a food supplement in Biologically Appropriate Raw Foods (BARF) diets for pets.

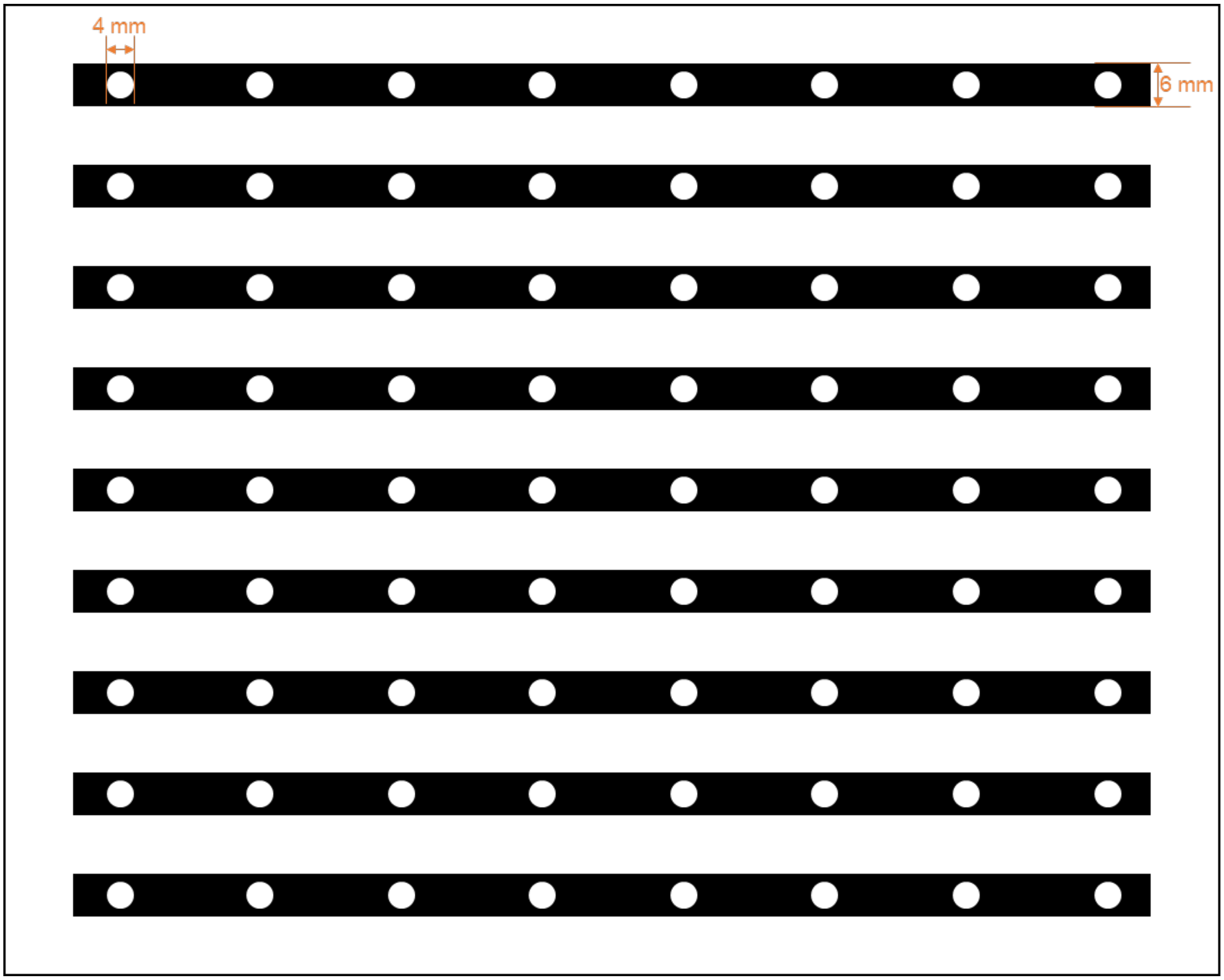

Figure S1. Wax pattern paper model for printing. The wax pattern was printed on Whatman No. 1 paper, with each spot initially having a diameter of 4 mm before undergoing thermal treatment. The total width of the pattern stripe is 6 mm. The use of the wax printer allowed for design flexibility, facilitating the creation of multispotted arrays. This design feature paves the way for potential automation of the sampling and analysis processes, ultimately enabling high throughput analysis.

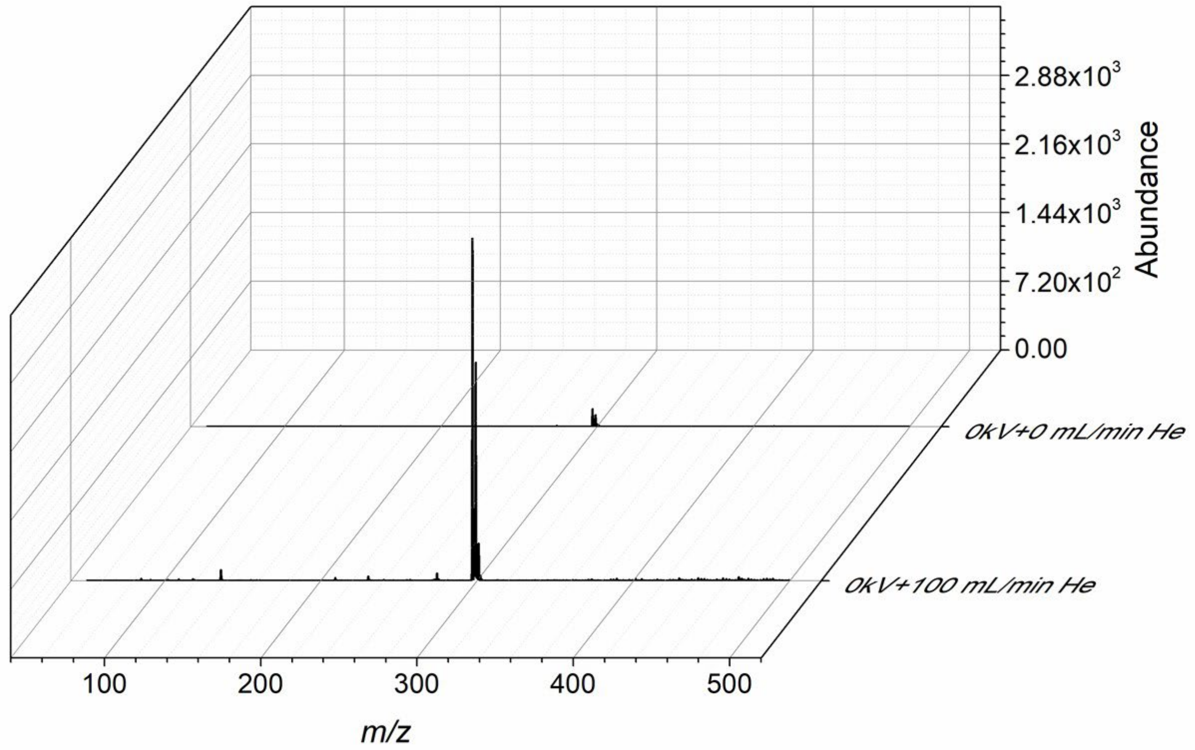

Figure S2. Mass spectrum depicting the ionization potential achieved through laser desorption. The influence of discharge gas presence was evaluated by comparing the absence (0 mL/min He) and presence (100 mL/min He) of gas flow. Remarkably, the utilization of gas flow resulted in a noticeable enhancement of signal intensity, facilitating the propulsion of analytes. However, it is important to note that the signal intensity observed in this method is three orders of magnitude lower compared to LD-FµTP-MS analysis.

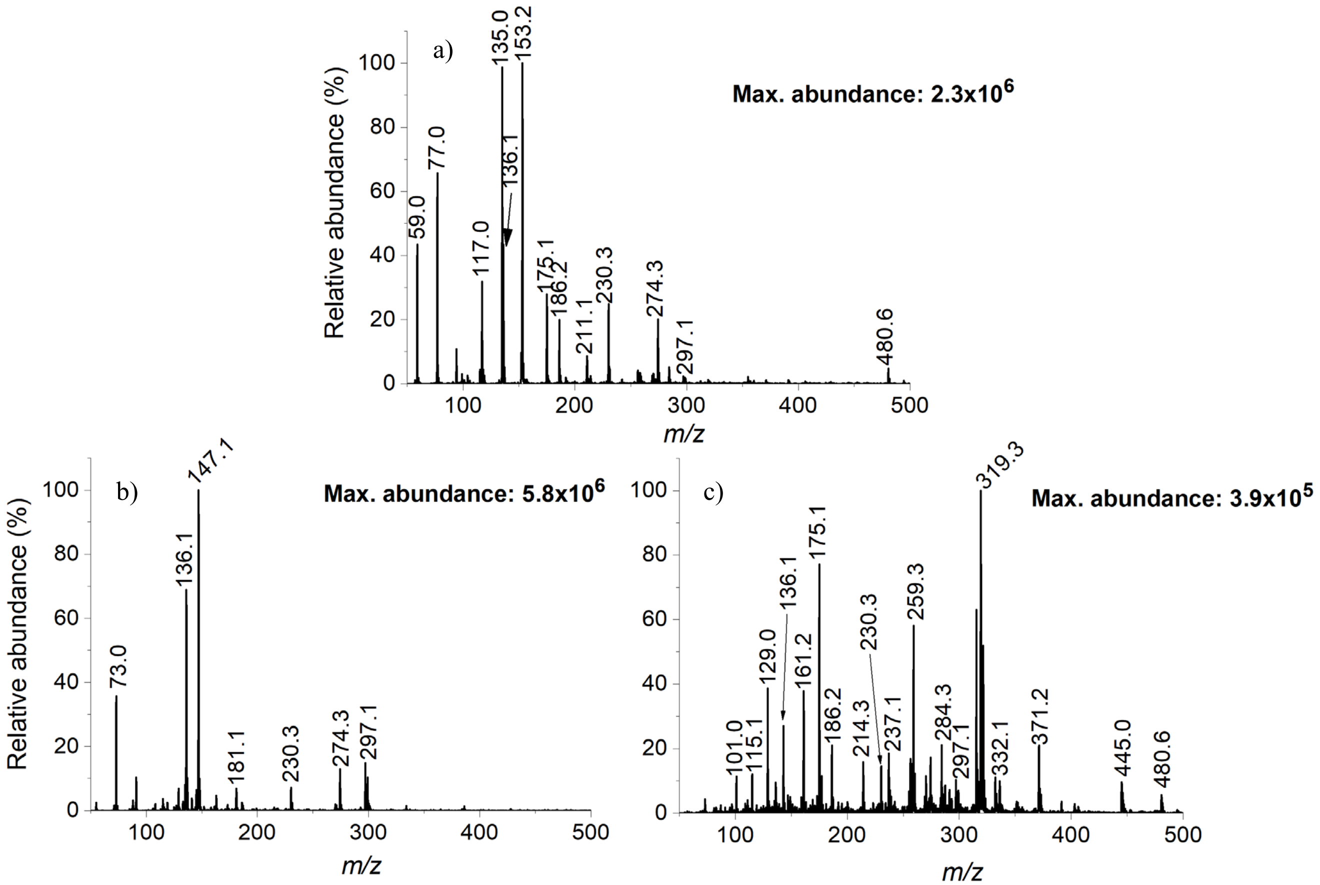

Figure S3. Mass spectra acquired through SALD-FμTP-MS analysis of the standard mixture sample deposited on white paper with graphite on the backside, utilizing different liquid matrices: a) propylene glycol (PG), b) butylene glycol (BG), and c) MeOH/water (1:1).

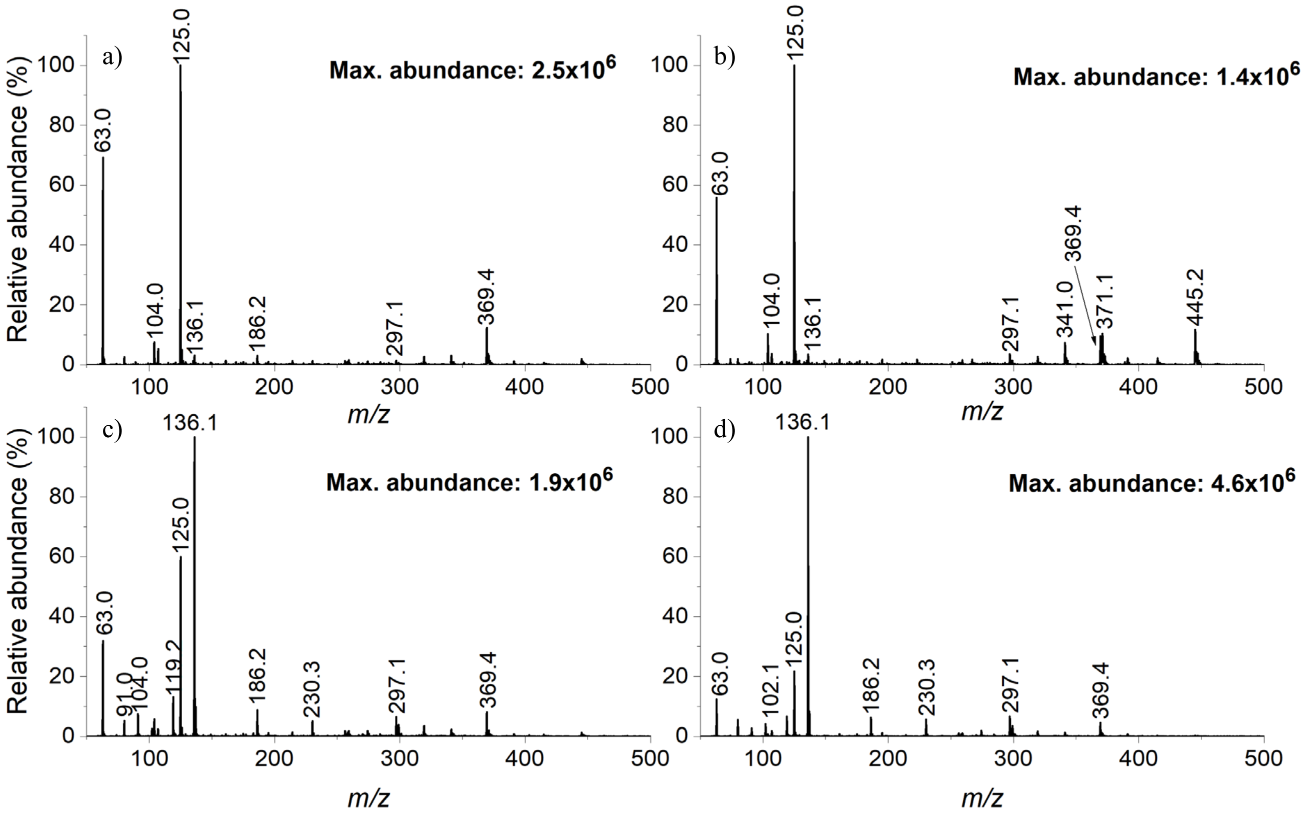

Figure S4. Mass spectra obtained from SALD-FμTP-MS analysis of the sample deposited on white paper with graphite on the backside using various liquid matrices: a) 25 mg/mL of 2,5-dihydroxybenzoic acid (2,5-DHB) dissolved in 60% ethylene glycol (EG) and 40% acetonitrile (ACN)/water (1:1), b) 25 mg/mL of α-cyano-4-hydroxycinnamic acid (CHCA) dissolved in 60% EG and 40% ACN/water (1:1), c) 25 mg/mL of 3-nitrobenzonitrile (3-NBN) dissolved in 60% EG and 40% ACN/water (1:1), and d) 60% EG and 40% ACN/water (1:1) without any MALDI matrix added.

The last figure is included to facilitate a comparison of the results when a MALDI matrix was used and optimum one.

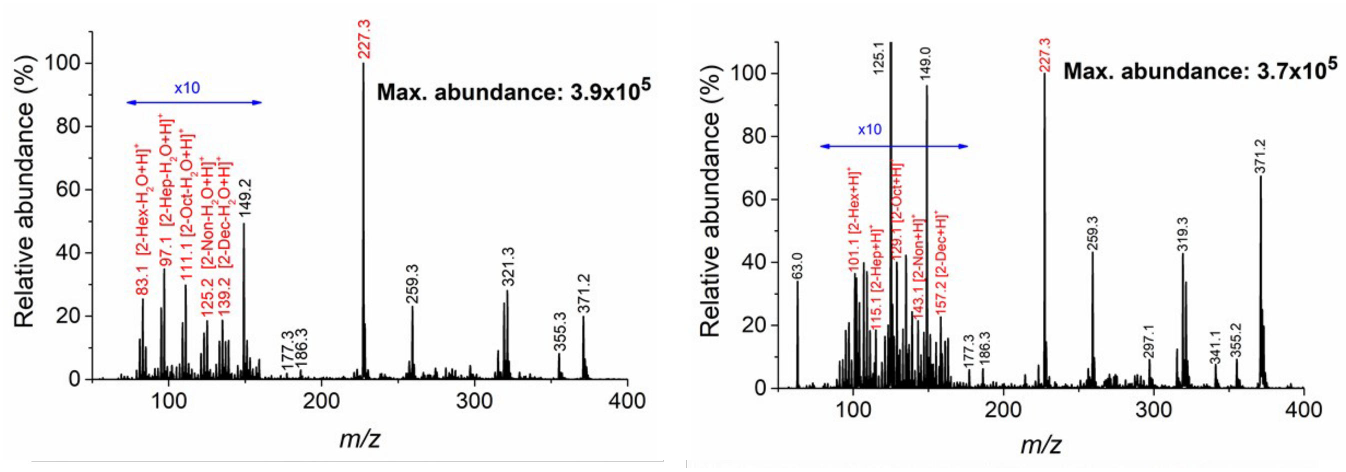

Figure S5. Impact of liquid matrix presence/absence on the fragmentation of the analytes using a mixture of seven ketones (2-hexanone, 2-heptanone, 2-octanone, 2-nonanone, 2-decanone, and 2-pentadecanone) at a concentration of 50 µM each in a MeOH/water (1:1) solution. a) SALD-FµTP-MS analysis without the liquid matrix, and b) SALD-FµTP-MS analysis with 60% ethylene glycol (EG) and 40% acetonitrile (ACN)/water (1:1) as the liquid matrix.

Table S1. Molecular weigth, *m/z* of the ions $[M+H]^+$ and $[M+H-H_2O]^+$, vapor pressure and boiling points of the seven evaluated ketones.

Ketone	Molecular weight	$[M+H]^+$	$[M+H-H_2O]^+$	Vapor pressure (mm Hg)	Boiling point (ºC)
2-hexanone	100.08882	101.0966	83.0861	11.6	127.6
2-heptanone	114.10447	115.1123	97.1018	3.85	151.5
2-octanone	128.12012	129.1279	111.1174	1.35	175
2-nonanone	142.13577	143.1436	125.1331	0.62	192
2-decanone	156.15142	157.1592	139.1487	0.25	210
2-pentadecanone	226.22967	227.2375	209.2266	0.003	293

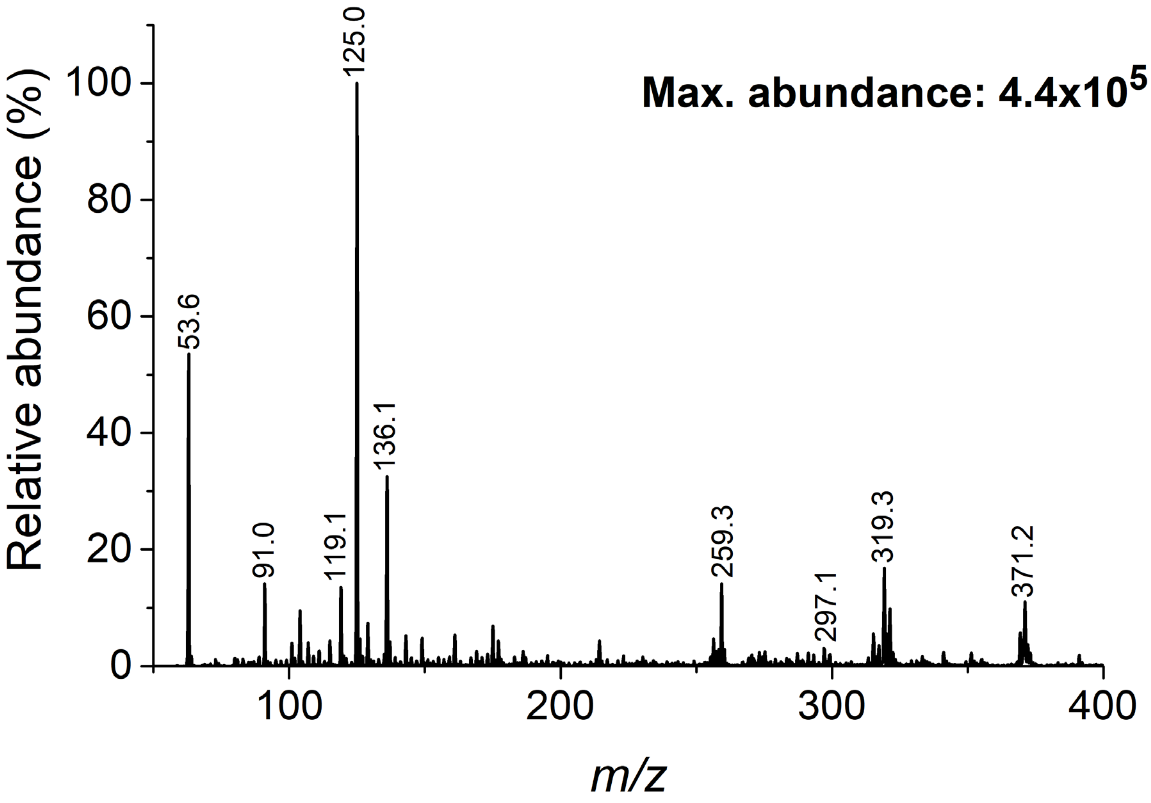

Figure S6. Mass spectrum obtained from SALD-FμTP-MS analysis in CP1 pseudo-continuous mode at 1.6 W using to desorb 250 picomoles of amphetamine, imazalil, and cholesterol deposited on patterned paper with graphite on the back of the paper. The liquid matrix utilized was a mixture of 60% EG and 40% ACN/water (1:1).

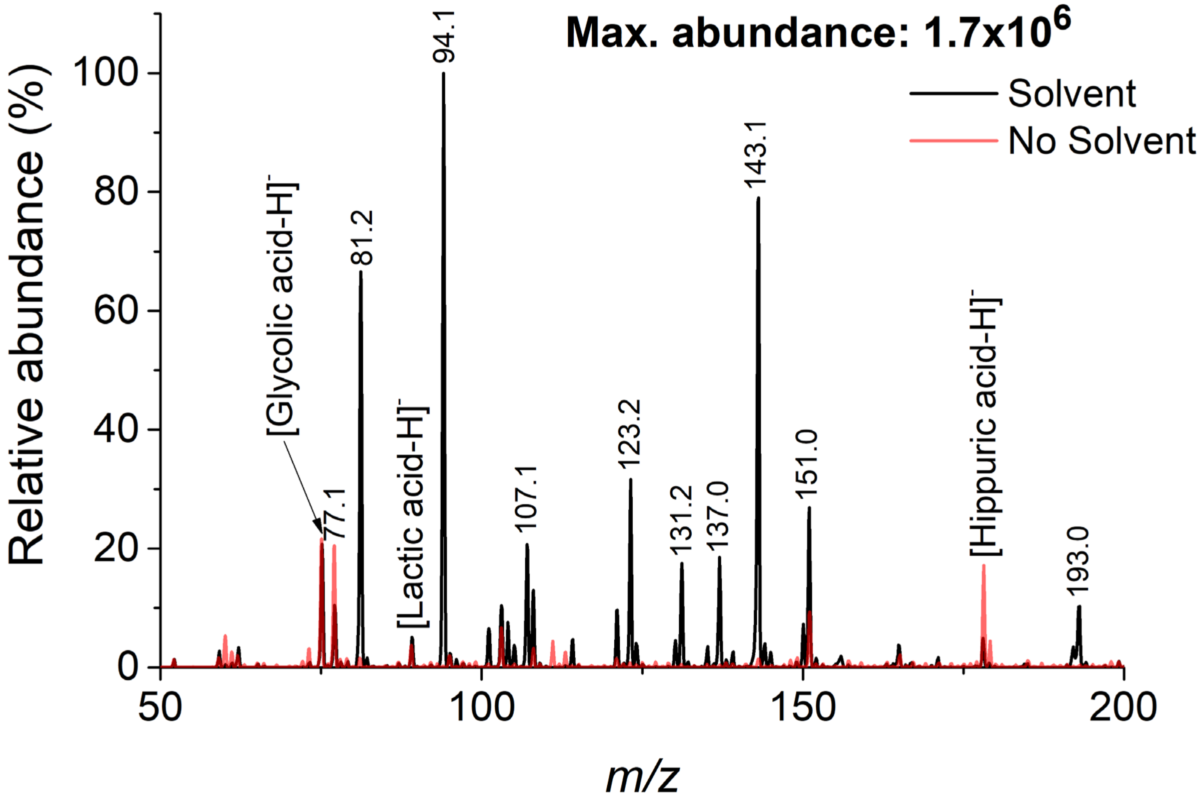

Figure S7. Mass spectrum obtained from SALD-FμTP-MS analysis in negative ion mode of 5 nanomoles of glycolic acid, lactic acid, and hippuric acid. The desorption process was performed using a liquid matrix composed of 60% EG and 40% ACN/water (1:1) (black trace), and without the liquid matrix (red trace).

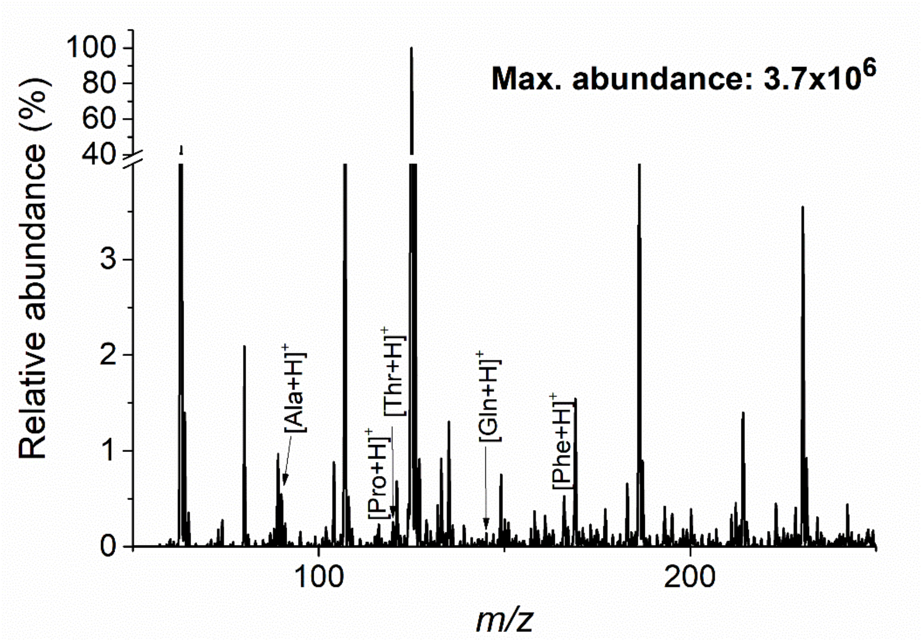

Compound	[M+H]+
Ala	90
Ser	106
Pro	116
Thr	120
Gln	147
Phe	166
Arg	175
Tyr	182

Figure S8. SALD-FµTP-MS analysis at the optimized conditions of 25 nanomoles of a mixture of 8 amino acids: alanine (Ala), serine (Ser), proline (Pro), threonine (Thr), glutamine (Gln), phenylalanine (Phe), arginine (Arg), and tyrosine (Tyr). The detected amino acids are highlighted in green, while the non-detected amino acids are highlighted in red in the side table.

Table S2. List of 31 pesticides spiked in the tap water sample and their corresponding mass spectrum in Figure 5a. The table includes highlighting for the detected compounds, compounds detected with low abundances, and potential isobars for the same *m/z*. The compounds in rows without highlighting indicate non-detected compounds.

Compound	Elemental composition (M)	$[M+H]^+$	$[M+H]^+$ Detected
Propoxur	$C_{11}H_{15}NO_3$	210.1125	210.17
Propachlor	$C_{11}H_{14}ClNO$	212.0837	212.08
Pymetrozine	$C_{10}H_{11}N_5O$	218.1036	218.15
Dichlorvos	$C_4H_7Cl_2PO_4$	220.9532	-
Chloridazon	$C_{10}H_8ClN_3O$	222.0429	-
Mepanipyrim	$C_{14}H_{13}N_3$	224.1182	224.15
Dicrotophos	$C_8H_{16}NPO_5$	238.0839	-
Oxadixyl	$C_{14}H_{18}N_2O_4$	279.1339	-
Myclobutanil	$C_{15}H_{17}ClN_4$	289.1215	289.33
Chloroxuron	$C_{15}H_{15}ClN_2O_2$	291.0895	-
Cyproconazole 1	$C_{15}H_{18}ClN_3O$	292.1211	-
Cyproconazole 2	$C_{15}H_{18}ClN_3O$	292.1211	-
Imazalil	$C_{14}H_{14}Cl_2N_2O$	297.0556	297.08
Fenpropimorph	$C_{20}H_{33}NO$	304.2635	304.33
Fenazaquin	$C_{20}H_{22}N_2O$	307.1805	307.17
Quinoxyfen	$C_{15}H_8Cl_2FNO$	308.004	-
Flusilazole	$C_{16}H_{15}F_2N_3Si$	316.1076	316.33
Bupirimate	$C_{13}H_{24}N_4O_3S$	317.1642	317.25
Diniconazole	$C_{15}H_{17}Cl_2N_3O$	326.0821	-
Pencycuron	$C_{19}H_{21}ClN_2O$	329.1415	329.21
Tebufenpyrad	$C_{18}H_{24}ClN_3O$	334.1681	337.23
Bitertanol	$C_{20}H_{23}N_3O_2$	338.1863	
Propiconazole	$C_{15}H_{17}Cl_2N_3O_2$	342.0771	342.08
Boscalid	$C_{18}H_{12}Cl_2N_2O$	343.0399	-
Tetrachlorvinphos	$C_{10}H_9Cl_4O_4P$	364.9065	-

Spiromesifen	$C_{23}H_{30}O_4$	371.2217	-
Profenofos	$C_{11}H_{15}BrClO_3PS$	372.9424	373.00
Proquinazid	$C_{14}H_{17}IN_2O_2$	373.0407	373.00
Bromuconazole 2	$C_{13}H_{12}BrCl_2N_3O$	375.9614	-
Fluquinconazole	$C_{16}H_8Cl_2FN_5O$	376.0163	376.08
Prochloraz	$C_{15}H_{16}Cl_3N_3O_2$	376.0381	376.08

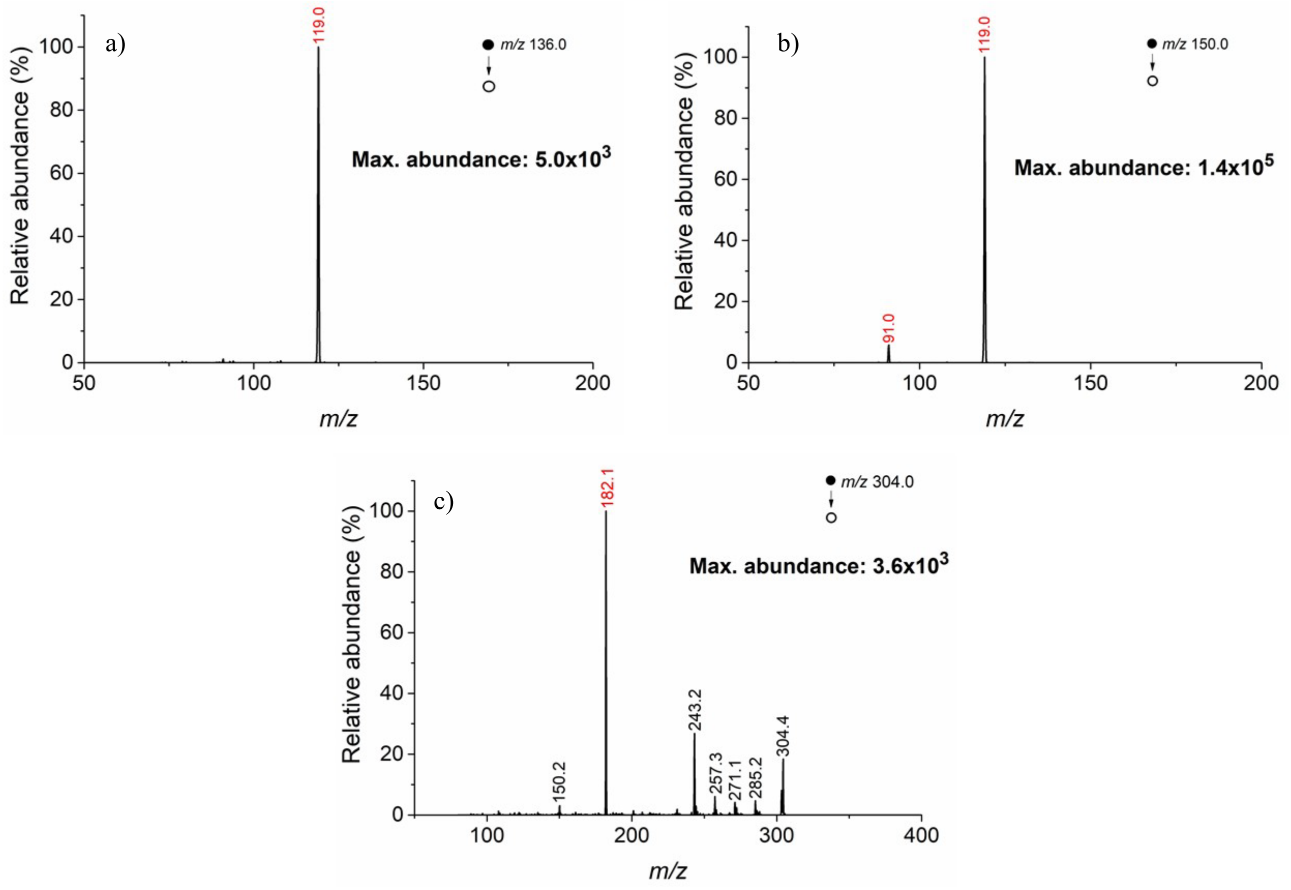

Figure S9. SALD-FμTP-MS Tandem MS mass spectra of the drugs spiked in the tap water sample collected in Figure 5b for a) amphetamine, b) methamphetamine, and c) cocaine. The analyzed compounds include:

a) Amphetamine:

Parent ion: *m/z* 136.0

Diagnostic fragment ion: *m/z* 119.0

b) Methamphetamine:

Parent ion: *m/z* 150.0

Diagnostic fragment ion: *m/z* 119.0

c) Cocaine:

Parent ion: *m/z* 304.0

Diagnostic fragment ion: *m/z* 182.1

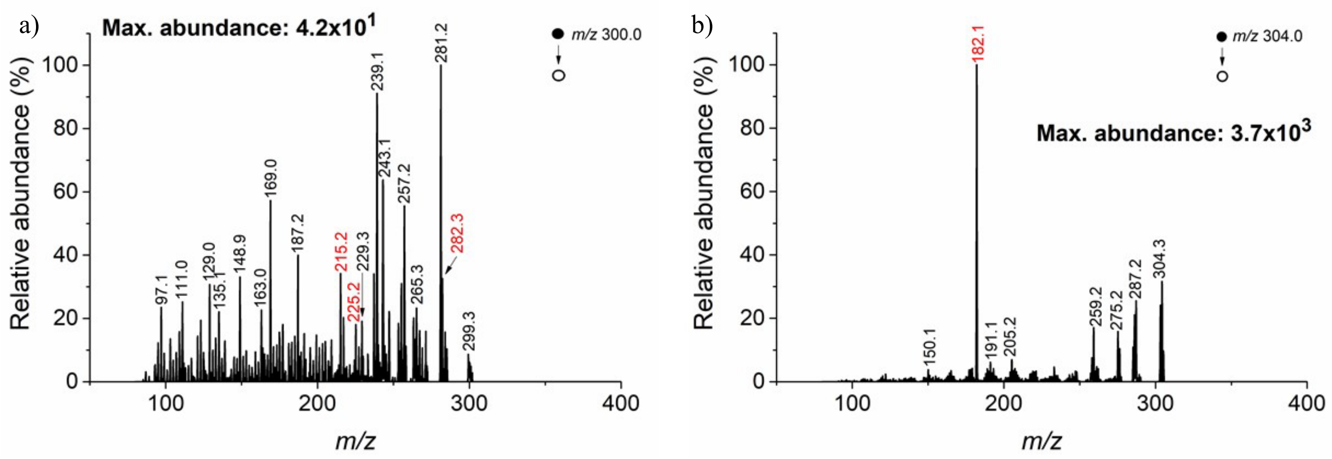

Figure S10. SALD-FuTP-MS Tandem MS mass spectra of a) 100 pg/µL codeine spiked in oral fluids, and b) 500 pg/µL cocaine soiked in human plasma.

a) 100 pg/µL of codeine spiked in oral fluid.

Parent ion: *m/z* 300.0

Diagnostic fragment ion: *m/z* 282.3, 225.2 and 215.2.

b) 500 pg/µL of cocaine spiked in human plasma.

Parent ion: *m/z* 304.0

Diagnostic fragment ion: *m/z* 182.1

Table S3. Candidates for tentative annotation of the *m/z* detected in bovine blood analysis as lipids. The candidates were determined using the LipidMaps annotation tool, considering the detected m/z values and selecting potential ion adducts [M+H]+ and [M+H-H2O]+, since the ionization was carried out qith a plasma-based ion source, within a delta window of ±0.1 Da.

Input *m/z*	Matched *m/z*	Delta	Name	Formula	Ion
369.42	369.3727	0.047	SFE 24:0	C24H48O2	$[M+H]^+$
369.42	369.3727	0.047	WE 24:0	C24H48O2	$[M+H]^+$
369.42	369.3727	0.047	FA 24:0	C24H48O2	$[M+H]^+$
369.42	369.3516	0.068	ST 27:1;O	C27H46O	$[M+H-H_2O]^+$
401.42	401.3778	0.042	ST 28:1;O	C28H48O	$[M+H]^+$
401.42	401.3625	0.058	MG O-21:1;O	C24H48O4	$[M+H]^+$
401.42	401.3778	0.042	ST 28:0;O2	C28H50O2	$[M+H-H_2O]^+$
401.42	401.3778	0.042	FA 28:3	C28H50O2	$[M+H-H_2O]^+$
488.33	488.3135	0.017	LPC 17:2	C25H48NO7P	$[M+H-H_2O]^+$
488.33	488.3135	0.017	LPE 20:2	C25H48NO7P	$[M+H-H_2O]^+$
488.33	488.3499	0.02	CerP 26:1;O2	C26H52NO6P	$[M+H-H_2O]^+$
488.33	488.3499	0.02	LPC O-18:2	C26H52NO6P	$[M+H-H_2O]^+$
516.42	516.4775	0.058	Cer 34:3;O2	C34H63NO3	$[M+H-H_2O]^+$
516.42	516.3448	0.075	LPE 22:2	C27H52NO7P	$[M+H-H_2O]^+$
544.5	544.5088	0.009	Cer 36:3;O2	C36H67NO3	$[M+H-H_2O]^+$
574.42	574.3867	0.033	PC 22:1	C30H58NO8P	$[M+H-H_2O]^+$
588.33	588.3296	4E-04	PE 24:3;O2	C29H52NO10P	$[M+H-H_2O]^+$
588.33	588.366	0.036	PC 22:2;O	C30H56NO9P	$[M+H-H_2O]^+$
588.33	588.4024	0.072	PE 26:1	C31H60NO8P	$[M+H-H_2O]^+$
602.33	602.3452	0.015	PE 25:3;O2	C30H54NO10P	$[M+H-H_2O]^+$
602.33	602.3816	0.052	PC 23:2;O	C31H58NO9P	$[M+H-H_2O]^+$
616.25	616.3245	0.075	PE 25:4;O3	C30H52NO11P	$[M+H-H_2O]^+$
644.42	644.3922	0.028	PC 25:3;O2	C33H60NO10P	$[M+H-H_2O]^+$
644.42	644.3922	0.028	PE 28:3;O2	C33H60NO10P	$[M+H-H_2O]^+$
644.42	644.465	0.045	PC 27:1	C35H68NO8P	$[M+H-H_2O]^+$
644.42	644.465	0.045	PE 30:1	C35H68NO8P	$[M+H-H_2O]^+$
658.42	658.4442	0.024	PE 30:3	C35H64NO8P	$[M+H]^+$
658.42	658.4078	0.012	PC 26:3;O2	C34H62NO10P	$[M+H-H_2O]^+$
658.42	658.4078	0.012	PE 29:3;O2	C34H62NO10P	$[M+H-H_2O]^+$
658.42	658.4078	0.012	PS 28:2	C34H62NO10P	$[M+H-H_2O]^+$

658.42	658.4442	0.024	PC 27:2;O	C35H66NO9P	$[M+H-H_2O]^+$
658.42	658.3715	0.049	PC 25:4;O3	C33H58NO11P	$[M+H-H_2O]^+$
658.42	658.4806	0.061	PC 28:1	C36H70NO8P	$[M+H-H_2O]^+$
658.42	658.4806	0.061	PE 31:1	C36H70NO8P	$[M+H-H_2O]^+$
714.33	714.3977	0.068	PS 30:4;O2	C36H62NO12P	$[M+H-H_2O]^+$
730.5	730.5017	0.002	PS O-33:3	C39H72NO9P	$[M+H]^+$
730.5	730.4654	0.035	PS 32:3	C38H68NO10P	$[M+H]^+$
730.5	730.4654	0.035	PC 30:4;O2	C38H68NO10P	$[M+H]^+$
730.5	730.5381	0.038	PE 35:2	C40H76NO8P	$[M+H]^+$
730.5	730.5381	0.038	PC 32:2	C40H76NO8P	$[M+H]^+$
730.5	730.5745	0.075	PE O-36:2	C41H80NO7P	$[M+H]^+$
730.5	730.5745	0.075	PC O-33:2	C41H80NO7P	$[M+H]^+$
730.5	730.5017	0.002	PS 33:1	C39H74NO10P	$[M+H-H_2O]^+$
730.5	730.517	0.017	PE O-38:7	C43H74NO7P	$[M+H-H_2O]^+$
730.5	730.4806	0.019	PE 37:7	C42H70NO8P	$[M+H-H_2O]^+$
730.5	730.4654	0.035	PC 30:3;O3	C38H70NO11P	$[M+H-H_2O]^+$
730.5	730.5381	0.038	PS O-34:1	C40H78NO9P	$[M+H-H_2O]^+$
730.5	730.5745	0.075	PC 33:0	C41H82NO8P	$[M+H-H_2O]^+$
730.5	730.5745	0.075	PE 36:0	C41H82NO8P	$[M+H-H_2O]^+$
758.5	758.4967	0.003	PS 34:3	C40H72NO10P	$[M+H]^+$
758.5	758.4755	0.025	PE 38:9	C43H68NO8P	$[M+H]^+$
758.5	758.533	0.033	PS O-35:3	C41H76NO9P	$[M+H]^+$
758.5	758.5694	0.069	PE 37:2	C42H80NO8P	$[M+H]^+$
758.5	758.5694	0.069	PC 34:2	C42H80NO8P	$[M+H]^+$
758.5	758.5119	0.012	PC 36:7	C44H74NO8P	$[M+H-H_2O]^+$
758.5	758.5119	0.012	PE 39:7	C44H74NO8P	$[M+H-H_2O]^+$
758.5	758.533	0.033	PS 35:1	C41H78NO10P	$[M+H-H_2O]^+$
758.5	758.5483	0.048	PE O-40:7	C45H78NO7P	$[M+H-H_2O]^+$
758.5	758.4391	0.061	PS 36:8	C42H66NO10P	$[M+H-H_2O]^+$
758.5	758.5694	0.069	PS O-36:1	C42H82NO9P	$[M+H-H_2O]^+$
786.5	786.5068	0.007	PE 40:9	C45H72NO8P	$[M+H]^+$
786.5	786.528	0.028	PS 36:3	C42H76NO10P	$[M+H]^+$
786.5	786.5643	0.064	PS O-37:3	C43H80NO9P	$[M+H]^+$
786.5	786.4704	0.03	PS 38:8	C44H70NO10P	$[M+H-H_2O]^+$
786.5	786.5432	0.043	PE 41:7	C46H78NO8P	$[M+H-H_2O]^+$

786.5	786.5432	0.043	PC 38:7	C46H78NO8P	$[M+H-H_2O]^+$
786.5	786.5643	0.064	PS 37:1	C43H82NO10P	$[M+H-H_2O]^+$
800.5	800.5225	0.023	PC 38:9	C46H74NO8P	$[M+H]^+$
800.5	800.5436	0.044	PS 37:3	C43H78NO10P	$[M+H]^+$
800.5	800.58	0.08	PS O-38:3	C44H82NO9P	$[M+H]^+$
800.5	800.4861	0.014	PS 39:8	C45H72NO10P	$[M+H-H_2O]^+$
800.5	800.5589	0.059	PC 39:7	C47H80NO8P	$[M+H-H_2O]^+$
800.5	800.5589	0.059	PE 42:7	C47H80NO8P	$[M+H-H_2O]^+$
